# Promoting aging migrants' capabilities: A randomized controlled trial concerning activities of daily living and self-rated health

**DOI:** 10.3934/publichealth.2018.2.173

**Published:** 2018-06-12

**Authors:** Emmelie Barenfeld, Synneve Dahlin-Ivanoff, Lars Wallin, Susanne Gustafsson

**Affiliations:** 1 Department of Health and Rehabilitation, Institute of Neuroscience and Physiology Sahlgrenska Academy, Centre for Ageing and Health—AgeCap, University of Gothenburg, Gothenburg, Sweden; 2 Department of Occupational therapy and Physiotherapy, The Sahlgrenska University Hospital, Gothenburg, Sweden; 3 School of Education, Health, and Social Studies, Dalarna University, Falun, Sweden; 4 Department of Neurobiology, Care Sciences and Society, Division of Nursing, Karolinska Institutet, Huddinge, Sweden; 5 Institute of Health and Care Sciences, The Sahlgrenska Academy, University of Gothenburg, Gothenburg, Sweden

**Keywords:** health promotion, person-centeredness, emigrants and immigrants, aged, capability

## Abstract

The aim was to evaluate the 6-month and 1-year effects of a person-centered group-based health-promoting intervention on independence in daily activities and self-rated health. The study was an RCT with follow-ups at 6 months and 1 year. A total of 131 independent living people (70+) who have migrated to Sweden from Finland or Western Balkan region were included. Participants were independent in activities of daily living and cognitively intact. They were randomized to an intervention group receiving four weekly group-meetings and a follow-up home visit, or a control group (no intervention). An overall chi-squared test was performed and the odds ratio calculated. A high proportion of the participants maintained independence in activities of daily living and improved or maintained self-rated health. However, no significant differences were found between the groups. The result indicates that the intervention was offered too early in the aging process to be able to detect effects. Methodological challenges were met during both the recruitment and implementation phases. In response to lessons learned, a multicenter design is recommended for future research in order to strengthen the findings. Furthermore, this study has contributed with experiences on both opportunities and challenges in terms of research with and about older people aging in the context of migration, as is discussed.

## 1. Introduction

There are requests from professionals about how to work in an evidence-based manner to reach out with health promotion to older people aging in the context of migration. This, since societal changes such as aging populations and global migration has seen an increasing number of people aging in the context of migration worldwide [1]. In Sweden, the proportion of older persons born abroad is currently increasing due to the country's migration history. Addtionally, aging persons might not have equal opportunities to enjoy good health. Individual factors, as well as factors at the group and societal level, form a basis for what a person actually can or cannot do to achieve desired health goals [2]. These factors are influenced by prior conditions in a person's country of birth, as well as in the current country of residence. This study reports results and lessons learned from evaluating a health promotion intervention in two Swedish city districts with low socioeconomic status for two of the largest immigrant groups among older people in Sweden; persons born in Finland or the Western Balkan region.

Health promotion interventions are one possible strategy to enable health in the aging population. In the health context, the term enablement refers to reducing differences in current health statuses and ensuring equal opportunities and resources to allow all people to achieve their *fullest health potential* [3]. In this paper health is defined as the ability to reach vital goals [4]. To maintain independence in daily activities has been expressed as a desired goal among older people and is also considered a means to promote health [5],[6]. Thus, a persons' ability to do what he/she wants to do is interrelated with their health [7]. Being dependent on others and losing control of one's effective performance of daily activities can be frustrating because they are related to one's personal identity, and feelings of independence in being able to perform desired activities whenever one wishes [8]. Dependence on others in daily activities may also negatively affect a person's capability, the effective possibilities a person has to utilize their resources to achieve their desired health goals [2].

Numerous studies and reviews on health promoting interventions in older people have found that interventions are important to support older people in managing their daily life and their experienced health [9]–[11]. Health promoting interventions should be offered prior to frailty [12], which is defined as a diminished ability to respond to stress resulting in vulnerability to poorer health outcomes [13],[14]. Older people aging in the context of migration are decribed to be at risk for both physical and social frailty. Aging may lead to decline in bodily functions [14]. In addition, psychosocial and cultural changes associated with migration can be experienced as stressful and can, as well as language problems, affect the life situation in the country of residence [15],[16]. Therefore, people aging in the context of migration are an important target group for health promotion. Previous studies have reported barriers related to accessing health services or health information among migrants [1],[15],[17]. Other studies have identified needs to adapt health promotion interventions to bridge cultural and linguistic barriers to make interventions available to the targeted population [18],[19]. However, to date, the evaluation of health-promotion interventions targeting older people aging in the context of migration has been scarce [18]. Furthermore, only two studies [20],[21] reported results concerning daily activities, however neither study showed significant findings. This knowledge gap suggests a need for further studies overall for health-related outcomes including daily activities.

Daily activities are doings intended for taking care of one's own body and supporting everyday life within the person's home and community [8]. In this study daily activities measures are defined as cleaning, shopping, transportation, cooking, bathing, dressing, going to the toilet, transfer, and feeding [22]. These measures constitute an appropriate primary outcome because of their defined significance for health and well-being [23]. Further, a subjective measure of health, *self-rated health*, can be an important additional outcome in effectiveness studies of health-promotion interventions. Self-rated health refers to the person's overall health and incorporates multiple subjective aspects of health that decline with age [24].

The group-based health promotion intervention *Senior Meetings* [25] has shown to prolong independence in daily activities and self-rated health among people 80 years or older in Sweden [11],[26]. However, it is unclear if the effect is maintained when the intervention is translated into practice in the everyday context of aging migrants. Thus, the intervention was further developed in a researcher community partnership from the existing evidence base [25] to welcome diversity. The adapted intervention “Promoting Aging Migrants Capabilities” (PAMC) [27] consists of four weekly group-sessions and an individual follow-up home visit. In line with existing research-evidence as how to perform health promotion for culturally and linguistically diverse older people, PAMC includes professional provision by an interprofessional team, activities, health information and a person-centered approach [18]. The hypothesis for the PAMC intervention was two-fold: (a) if a health-promoting intervention is introduced when older persons who were born abroad are pre-frail, it is possible to prevent or delay both deterioration in health (i.e. dependence in ADL, self-rated health) and life satisfaction; (b) the design and content of the evidence-based *Senior Meetings* can be used in the context of older migrants living in Sweden. A feasibility study of PAMC [28] showed promising results by indicating that the intervention bridged barriers to health-promotion services. Further, PAMC has in qualitative studies shown to raise awareness of one's health promoting behavior in past, present, and later life [29], and to support decision-making to satisfy health needs of self or others in everyday life [30]. However, it is not known whether this adapted health promoting intervention can positively impact independence in daily activities and self-rated health. Accordingly, the present study aimed to evaluate the 6-month and 1-year effects of PAMC concerning independence in daily activities and self-rated health.

## 2. Materials and methods

### 2.1. Study design and context

PAMC was a pragmatic randomized controlled trial [31] consisting of one intervention group and one control group. The trial addressed independent living people ≥ 70 years old who had migrated to Sweden from Finland or the Western Balkan region. Details of the study design have been reported elsewhere [27]. The present study concerns the 6-month and 1-year follow-ups conducted 2012–2016. The Regional Ethical Review Board approved the study (#821-11), and written informed consent was obtained from all participants. The trial is registered at ClinicalTrials.gov (NCT01841853).

#### 2.1.1. Participants

**Figure 1.**
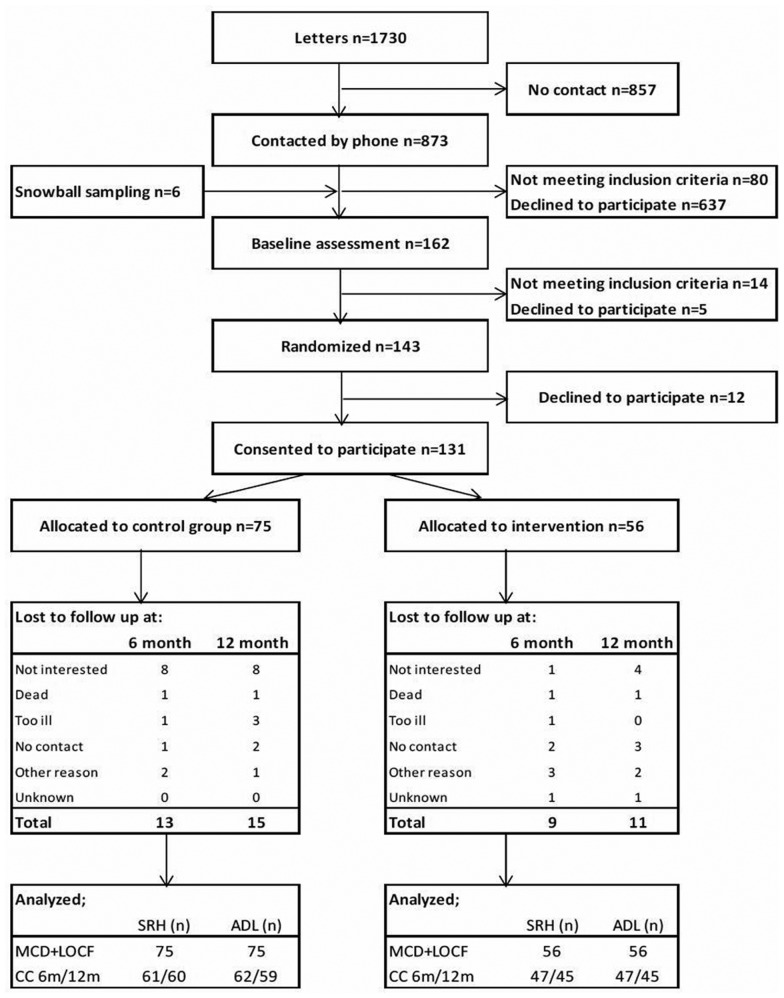
The flow of participants through the Promoting Aging Migrants' Capabilities study and the reason for declining participation at the 6-month and 1-year follow ups.

Enrollment of participants took place in three waves of recruitment. In the first wave, those eligible to participate were drawn from official registers in one selected suburban district of a medium-sized Swedish city. As the targeted inclusion rate was not achieved in the first wave, the same procedure was repeated in a second wave in another suburban area with similar demographics. Snowball sampling [32] was used for the third wave to reach the targeted inclusion rate. Further details of the recruitment process can be found at Gustafsson et al. [27]. The following inclusion criteria were applied: (a) migrated to Sweden from Finland or the Balkan Peninsula; (b) ≥ 70 years old; and (c) community-dwelling and independent of help from another person in activities of daily living, as measured by the ADL-staircase [22],[33]. Thus, for pragmatic reasons the study included persons speaking Finnish, Bosnian/Croatian/Serbian (BCS) or Swedish. Impaired cognition was an exclusion criterion. Therefore, those who scored < 80% of administrated items on the Mini-Mental State Examination [34] at baseline were excluded and referred to appropriate health care services. In total, 131 persons fulfilled the inclusion criteria and consented to participate (Figure 1). The majority of allocated participants were recruited in the first wave (n = 88) while the second and third waves added 37 and 6 participants, respectively.

#### 2.1.2. Procedure

The three recruitment waves had differing enrollment steps that preceded assessment of eligibility. In the first and second waves, letters were sent by postal mail (n = 1730), followed by a telephone call approximately 1–2 weeks later. If no telephone number was available, a second letter was sent with a request for a response. In the third wave, information about the study was disseminated by former participants or key persons in the reference groups who had been involved in intervention development, and by advertising on a local radio station. Those interested in participating were asked to make contact with the research team for more information. People, with whom contact was established by telephone or post, were requested to participate (n = 873) (Figure 1). Trained research assistants conducted the enrollment and data collection in the participants' preferred language.

The health-promotion intervention was conducted in two suburban districts conveniently located for the first and second recruitment waves. The first and second recruitment waves targeted districts with a large proportion of residents who were born abroad and with a generally low income and educational level. In the third wave participants from other districts in the medium-sized city were also included. See Table 1 for demographic details of the suburban districts, the medium-sized city and Sweden.

**Table 1.** Overview of the demographics of the study settings.

Demographics	Suburban area 1	Suburban area 2	Medium-sized city	Sweden
Total population 2015	51214	48274	548190	9851017
Born abroad	51%	42%	23%	16%
Aged ≥ 65 years	11%	12%	15%	20%
General education level^1^	10%	11%	24%	28%
General income level^2^				
Swedish krona	176700	180600	243400	228400
Euro^3^	17200	17500	23700	22200
USD^3^	20150	20600	27800	26000

Note: ^1^University ≥ 3 years among people aged 65–74 years; ^2^For people aged ≥ 65 years; ^3^Approximate value.

#### 2.1.3. Intervention group

The intervention comprised four weekly group meetings (4–6 participants) and one follow-up home visit. The group meetings were based on discussions originating from a booklet which the participants could read or listen to in advance. The booklet covered different aspects of self-management of health such as physical activity, medication, nutrition, assistive devices, adaptation of housing, memory and quality of life. See Gustafsson et al. [26] for further details. Health information was also provided orally and further discussed during the meetings. A multidisciplinary team consisting of an occupational therapist, a physiotherapist, a registered nurse, and a qualified social worker administered the meetings, with team members responsible for one session each. One team member, the group leader, followed each set of group meetings with the goals of establishing continuity and stimulating group processes. In line with seeking a person-centered approach, which emphasizes people's expertise regarding their own situations [35],[36], group discussions during the group meetings varied in accordance with the participants' experiences, needs, and resources. Peer-learning [37] was also used to enable participants to learn from and support each other. About 2 weeks after the group meetings were finished, the participants were offered an individual follow-up home visit conducted by one of the professional team members. The home visit entailed possibilities to clarify any content from the group meetings as well as deepening the information of areas of personal interest.

A bilingual approach was used wherein participants could choose their preferred language of communication. An interpreter was included in the intervention team when needed and interpretation was used according to preferences within the group. Hence, meetings or follow-up home visits could be held in Swedish, the participants' mother tongue, or a combination of both.

#### 2.1.4. Control group

The participants allocated to the control group received no intervention. However, they could, on their own initiative, approach the usual range of community or health services (e.g., home help services, rehabilitation, or medical care). If an urgent need for community or health care services was identified at baseline or at follow ups, information was provided on where to receive help.

### 2.2. Assessments and outcomes

Assessments were conducted in the participants' own homes or in an alternative location if the participant wished. The personnel in the inter-professional team or research assistants performed baseline assessments. Research assistants performed the follow-up assessments.

#### 2.2.1. Activities of daily living

The primary outcome, independence from, or dependence on, another person in daily activities was assessed based on the ADL-staircase [22],[33], via interview. The ADL-staircase applies a cumulative scale of well-defined personal and instrumental activities. In this study, nine out of the 10 original activities in the ADL-staircase were used; cleaning, shopping, transportation, cooking, bathing, dressing, going to the toilet, transfer, and feeding. Change between baseline and follow-up were dichotomized into either maintained independence in all activities or dependence in one or more activities. Dependence was defined as receiving personal or directive assistance from another person. Participants living with another person were assessed as “independent” if they were capable of performing the activity independently.

#### 2.2.2. Self-rated health

The secondary outcome, self-rated health, was measured by the first question in the 36-Item Short Form Survey (SF-36) [38], “In general, you would say your health is…” Response choices were: (1) excellent; (2) very good; (3) good; (4) fair; or (5) bad. The changes between baseline and respective follow-up were dichotomized into either decreased or maintained/improved.

### 2.3. Sample size, randomization, and blinding

A power calculation was made based on an outcome variable in the PAMC study—the Berg Balance Scale [39]. This showed that 65 participants in each study arm was needed to achieve a power of 80% at a significance level of α = 0.05 for detecting a difference of ≥ 15% between the study arms.

The randomization *was* stratified to enroll an equal amount of people from Finland and the Western Balkan region. An independent researcher, not involved in enrolling participants or delivering the intervention, organized the allocation system. Opaque, sealed envelopes were used to randomly assign participants to either the control or intervention group. Randomization was performed after baseline assessment. To enable blinding of those assessing the outcomes for group assignment, the intent was that different parties conducted baseline assessments and follow-ups. This intent was met for a majority of the participants.

### 2.4. Statistical analysis

Both descriptive and analytical statistics were used to compare groups and for analysis of change over time. The number of participants who had maintained independence in daily activities or maintained or improved self-rated health was calculated during the course of the study using the measures described above. In line with the hypothesis, the participants were dichotomized into maintained/improved or non-maintained from baseline to respective follow-up in the final analysis. An overall chi-squared test was performed to test differences in the proportions of participants who maintained/improved at follow-ups. Thereafter, the odds ratio (OR) was calculated in order to compare the groups. All statistical tests were two-sided, with *p* < 0.05 considered statistically significant. The confidence interval (CI) was calculated, using normal approximation of the log-odds ratio. Data were analyzed using SPSS version 22 (IBM Corp., Armonk, NY, 2009).

Analyses were performed based on the intention-to-treat principle, which entailed that all participants were analyzed in the group to which they were randomized [40]. To present as nuanced and comprehensive result as possible, analysis were conducted by using two different imputation methods for those lost to follow-up; the median change of deterioration (MCD) [41] and the last observation carried forward (LOCF) [42]. In addition, a complete case analysis (CC) was performed, serving as a sensitivity analysis. The assumptions for MCD as an imputation method were that older people are expected to deteriorate in health over time due to the aging process, and that deteriorated health is a common reason for not completing the follow-ups [43]. Thus, missing values were replaced with a value based on the median change of deterioration between baseline and follow-ups. In contrast, LOCF were based on assumptions that the average unobserved outcomes within each randomized group do not change over time, meaning that health and independence were considered to be maintained [42]. Using this approach, missing values at follow-ups were replaced with the last known observed value either from baseline or 6-month follow-up. Values for worst-case change were imputed for people who had died before follow-up.

## 3. Results

Baseline assessments were performed for 162 people, and 131 satisfied the inclusion criteria and consented to participate. Participants were randomized August 2012–December 2014: 75 were allocated to the control group and 56 to the intervention group (Figure 1). Baseline demographics showed no significant differences between the groups (Table 2).

**Table 2.** Baseline characteristics of demographic variables and outcome measures.

	Total	Intervention	Control
	n = 131	n = 56	n = 75
Age range	70–84	70–82	70–84
(mean, SD)	(74.1, SD 3.4)	(74.0, SD 3.4)	(74.1, SD 3.4)
Male, n (%)	66 (50)	37 (49)	29 (52)
Living alone, n (%)	63 (48)	31 (55)	32 (43)
Type of housing, n (%)			
*Tenant*	68 (52)	30 (54)	38 (51)
*Owner of apartment*	26 (20)	9 (16)	17 (23)
*Owner of house*	35 (27)	16 (28)	19 (25)
*Other*	2 (1.5)	1 (2)	1 (1)
Education, n (%)			
*Tertiary education^1^*	20 (16)	8 (14)	12 (16)
*Low education^2^*	28 (22)	15 (27)	13 (18)
Migrated from, n (%)			
*Western Balkan region*	60 (46)	22 (39)	38 (51)
*Finland*	71 (54)	34 (61)	37 (49)
Years lived in Sweden, n (%)			
≥ 21 years	114 (87)	51 (91)	63 (84)
Reasons for migration, n (%)			
*Labor*	47 (37)	27 (50)	20 (27)
*Refugee*	26 (20)	9 (17)	17 (23)
*Family*	16 (13)	7 (13)	9 (12)
*Other*	38 (30)	11 (20)	27 (37)
Good self-rated overall ability to speak Swedish, n (%)	117 (89)	51 (91)	66 (88)
Good self-rated overall ability to speak Swedish in contact with authorities, n (%)	103 (79)	44 (79)	59 (79)
Satisfied with economic situation^3^ n (%)	74 (56)	30 (54)	44 (60)
Independent in ADL	131 (100)	56 (100)	75 (100)
Health rated as good or better^4^	89 (68)	35 (63)	54 (72)

Note: ^1^Tertiary education (university > 3 years). ^2^Low education (elementary school or no education). ^3^Satisfied with economic situation rated with LiSat [44]. ^4^Self-rated Health measured by the first question in the 36-Item Short Form Survey (SF-36) [38].

The dropout rate at the 6-month and 1-year follow-ups were, respectively, 17% (n = 22) and 20% (n = 26). No significant differences were found between the dropout rates in the intervention and control groups (6-month *p* = 0.848, 1-year *p* = 0.960). “Not interested” was the main reason for declining participation at both the 6-month and 1-year follow-up, and further reasons are provided in Figure 1.

No significant differences were found between participants and dropouts with regard to age, living conditions, educational level, reasons for migration, or years lived in Sweden. Neither were differences found for self-rated health, decreased balance, hand strength or gait speed (*p* > 0.05). However, a significantly higher proportion of dropouts compared to participants were found for the variables: Male and being born in the Western Balkan region. Additionally, among dropouts, there was a lower proportion of people who scored > 25 on the MMSE [34], who were single living, and rated their ability to speak Swedish when in contact with authorities as good when compared with remaining participants. Table 3 displays the variables demonstrating significant differences between dropouts and non-dropouts.

**Table 3.** Variables showing significant differences in proportion of dropouts and participants at the 6-month and/or the 1-year follow-ups.

	6-months			1-year		
	Dropouts	Participants	*p*-value	Dropouts	Participants	*p*-value
	n = 22 (%)	n = 109 (%)		n = 26 (%)	n = 105 (%)	
Sex (male)	73	46	0.022	65	47	0.087
Living alone	27	52	0.032	35	51	0.124
Migrated from the Western Balkan region	82	39	0.000	81	37	0.000
Good self-rated overall ability to speak Swedish when in contact with authorities	68	81	0.190	62	83	0.018
Mini-Mental State Examination > 25 points	77	96	0.007	77	97	0.002

The intervention was implemented in accordance with the protocol. Fifty-seven percent of the participants in the intervention group (n = 32) attended all four meetings, while 16% (n = 9) attended three meetings, 9% (n = 5) two meetings, and 11% (n = 6) one meeting only. Four people attended no meetings. No adverse events were reported during implementation of the intervention.

In line with the inclusion criteria, all participants were independent in ADL at the study baseline. Their self-rated health varied from poor to excellent with a median value reflecting good health for both groups. A higher proportion in the control group rated their health as good or better at baseline when compared to the intervention group (72% vs. 63%, see Table 2), however, this difference was not statistically significant (*p* = 0.263). No significant differences were found between the intervention and control group regarding maintenance in ADL or maintenance/improvement in self-rated health at follow-ups. All three analysis methods showed that a high proportion of the participants in both groups maintained independence in ADL at the 6-month and 1-year follow ups, although the proportion did depend on the method used (Table 4). The same pattern was shown for self-rated health (Table 5).

**Table 4.** Proportion (%), odds ratio (OR), 95% confidence interval (CI), and *p*-value for maintained independence in activities of daily living at 6 months and 1 year between control group and intervention group presented for complete case analysis and two different imputation methods.

Analysis method	Maintained independence in ADL at follow-up	Control group^1^ n (%)	Intervention group n (%)	OR	CI (OR)	*p*
MCD						
n = 131	6 months	53 (71)	42 (75)	1.25	0.57–2.72	0.583
	1 year	54 (72)	38 (68)	0.82	0.39–1.75	0.608
LOCF						
n = 131	6 months	65 (87)	50 (89)	1.28	0.44–3.76	0.651
	1 year	67 (89)	48 (86)	0.72	0.25–2.04	0.533
CC						
n = 109^2^	6 months	53 (85)	42 (89)	1.43	0.44–4.58	0.550
n = 104^3^	1 year	54 (92)	38 (84)	0.5	0.15–1.7	0.269

Note:^1^Reference group (1.00); ^2^control n = 62, intervention n = 47; ^3^control n = 59, intervention n = 45.

**Table 5.** Proportion (%), odds ratio (OR), 95% confidence interval (CI), and *p*-value for maintenance or improvement in self-rated health at 6 months and 1 year between control group and intervention group presented for complete case analysis and two different imputation methods.

Analysis method	Maintenance or improvement in self-rated health at follow-up	Control group^1^ n (%)	Intervention group n (%)	OR	CI (OR)	*p*
MCD						
n = 131	6 months	42 (56)	33 (59)	1.13	0.56–2.27	0.738
	1 year	38 (51)	30 (54)	1.12	0.56–2.25	0.742
LOCF						
n = 131	6 months	55 (73)	40 (71)	0.91	0.42–1.97	0.809
	1 year	51 (68)	38 (68)	0.99	0.47–2.09	0.986
CC						
n = 108^2^	6 months	42 (69)	32 (68)	0.97	0.43–2.19	0.932
n = 105^3^	1 year	37 (62)	28 (62)	1.02	0.46–2.27	0.954

Note: ^1^Reference group (1.00); ^2^control group n = 61, intervention n = 47; ^3^control group n = 60, intervention n = 45.

## 4. Discussion and conclusions

The intervention demonstrated no significant results for either independence in daily activities or self-rated health, neither at 6 months, nor 1-year follow-up. One possible explanation for this is that the intervention had no effect, but ‘lessons learned’ during the recruitment and implementation phases of PAMC indicate that there may be further explanations. These ‘lessons learned’ can be used to guide the design and conduct of future research, and contribute to the discussion of if, when, and to whom the intervention should be offered.

A main factor contributing to the difficulty of concluding the effect of the intervention is that of the power in PAMC. The power analysis determined the number of participants to be a total of 130, with 65 in each study arm [26]. After an extended recruitment procedure, wherein all three planned waves for recruitment had to be applied, 131 participants were allocated to each of the two study arms. However, due in part to more participants who were randomized to the intervention group declining participation (Figure 1), an imbalance emerged in the distribution of participants between the study arms at the expense of the intervention group (n = 56) when in comparison to the control group (n = 75). Notwithstanding this imbalance, there was no difference in demographics between the intervention and control group (Table 2). Clearly, however, an insufficient number of participants in the intervention group may have influenced the result. Based on this lesson learned regarding power, we recommend future similar studies to consider a multi-center design to ensure a sufficient number of participants.

One main finding in this study was that a high proportion of participants in both the intervention and control group maintained independence in ADL. This indicated that the intervention might have been offered too early in the aging process to be able to show an effect through the selected outcome-measures. Considering the shift to more preventive and promotive health services, there is a need of sensitive measures to capture longitudinal changes among people who are still independent in daily activities. The measure used for self-rated health, that is the first question in the SF-36 [38], is widely used in research and has been proven successful in acquiring a comprehensive picture of an older individual's health [21],[24]. However, there may be better alternatives for the particular target group in the PAMC study, as is shown by our qualitative process evaluations [29],[30]. One such option may be a measure of quality of life, including aspects of health but focused on an individuals' abilities to “do” and “be” what they consider important in life; e.g., the ICEpop CAPability measure for Older people (ICECAP-O) [45],[46]. Concerning the ADL-staircase [22],[33], the dichotomous outcome of the nine activities (dependence/independence) may, similar to most available ADL-ability outcome-instrument, be too approximate of a measure. Additional activity dimensions, such as feeling secure when performing them or performing them without fatigue or adding leisure activities, may be more sensitive outcomes for the target group.

The assumption that older people aging in a migration context might be doubly vulnerable and more prone to ill-health compared with their native-born counterparts was based on available research [15],[47],[48] and guided the decision to lower the target age for participating in the PAMC study as compared to the age in the original study [25],[49]. It is possible that older migrants from the targeted group have more resources, within the person and his/her environment, and are less vulnerable than previously thought. However, another explanation could be that we did not succeed to reach the people that would have benefitted most from the intervention. Participants' characteristics (Table 2) show that heterogeneity in the PAMC intervention was reached due to migration experiences, but it should be noted that most participants have been living in Sweden for more than 20 years. In addition, educational level and income are described as social determinants for health [3], and are important to consider as this study was performed in an area with an overall lower educational level and socio-economic status. However, the results showed that people with poor language skills and people with lower levels of education were most likely not included in the PAMC sample. There were also a significantly higher proportion of drop-outs at 12 months who rating their overall ability to speak Swedish in contact with authorities as “low” when compared to persons who continued to participate. Lower scores on MMSE [34] among drop-outs also indicated that people with mild cognitive decline and poorer language skills at baseline may have been lost to follow up (Table 3). This may have affected the results, and calls for further studies on motives for participation and how to reach and enhance recruitment rates for people at highest risk of physical and social frailty.

The fact that a significantly higher proportion of drop-outs in participants born in the Western Balkan region than Finland at both follow-ups (Table 3) needs to be further discussed. In addressing the target group, i.e. older persons born abroad, the intention was that the study group should comprise a sample as representative as possible of older persons in the urban district who had migrated to Sweden. Thus, the two largest groups of immigrants were chosen; persons born in Finland and persons born in the Western Balkan region [27]. This pragmatic approach can be seen as advantageous considering the possibility of implementing the results in practice and the fact that previous studies in the Swedish context often have included persons from a single country. Frailty and health are affected by health determinants both in the country of birth and the country of residence after migration [13],[47]. Thus, considering the arisen imbalance, it could also be seen as a disadvantageous approach. This, since we did not separate persons born in different countries in the analysis despite possible differences between these groups, but saw them as a cohesive group in a person-centered manner. Even if the use of MCD as an imputation method to some extent may have compensated for this in the analysis, there is a possibility that the imbalance among drop-outs concerning country of birth has affected the analysis of the outcome of the intervention.

A further possible limitation in the design of the PAMC study is the risk of spillover effect. In the first two waves of the recruitment process, two urban districts were approached and people ≥ 70 years old born in Finland or the Western Balkan region were invited to participate. Although this target group constitutes two of the largest groups of older migrants in the respective urban districts, their relative numbers are small and the likelihood of numerous residents from these groups already knowing each other was high. Consequently, there was a risk of a spillover effect in that members of the intervention group shared their experiences and acquired knowledge with members of the control group. This suspicion was amplified when considering the results from qualitative process evaluations [29],[30]. Members of the intervention group expressed that they were seeking and using health information not only to improve their own health, but also to support relatives and friends to satisfy their health needs. While this finding from the qualitative evaluations, from a public health perspective, must be perceived as positive, it might have contributed to the null-results in the present study.

The conduct of this study has contributed with lessons learned on both opportunities and challenges in terms of research with and about older people aging in the context of migration. Immigrants or people with low socioeconomic backgrounds are often underrepresented or excluded in research designs and from participating in trials [50],[51]. Methodological challenges were met in the recruitment procedure, language barriers in data collection and including a heterogeneous population. These challenges might have influenced the results and may therefore be seen as methodological limitations. However, by actively dealing with such methodological challenges our study contributed to a better understanding of the diverse needs of older populations in research and practice.

To conclude, no intervention effect was demonstrated concerning independence in daily activities and self-rated health. Several possible explanations were discussed such as choice of outcome measures, challenges in recruitment, the timing of the intervention and the risk of spillover effect which might have influenced the findings. However, the fact that power in the analysis was not reached is considered to be the most likely explanation as to why no intervention effect was detected. Before a final conclusion on the intervention impact, and for whom it is effective can be determined, further studies are needed. This study showed that a high proportion of the participants in both groups maintained their independence in ADL and self-rated health, which indicates that the intervention was offered too early during the aging process to be able to impact on the used outcome measures. Taking the heterogeneity of people in the target group into account, a more person-centered approach with, for instance screening of frailty [52] in combination with people's own opinions about their need for lifestyle changes may be a way to identify persons who would benefit most of the intervention. The above-mentioned lessons learned should be taken into consideration when designing research and interventions targeting older persons aging in the context of migration. Further on, a multicenter design is recommended for future studies as this might address discussed methodological challenges.
